# Venous congestion affects neuromuscular changes in pigs in terms of muscle electrical activity and muscle stiffness

**DOI:** 10.1371/journal.pone.0289266

**Published:** 2023-08-03

**Authors:** Keun-Tae Kim, Duguma T. Gemechu, Eunyoung Seo, Taehoon Lee, Jong Woong Park, Inchan Youn, Jong Woo Kang, Song Joo Lee

**Affiliations:** 1 Korea Institute of Science and Technology, Bionics Research Center, Seoul, Korea Repub; 2 Division of Bio-Medical Science & Technology, KIST School, Korea University of Science and Technology, Seoul, Korea Repub; 3 Department of Orthopaedic Surgery, Korea University Ansan Hospital, Ansan, Korea Repub; 4 Department of Orthopaedic Surgery, Korea University Anam Hospital, Seoul, Korea Repub; University of Illinois at Urbana-Champaign, UNITED STATES

## Abstract

Early detection of venous congestion (VC)-related diseases such as deep vein thrombosis (DVT) is important to prevent irreversible or serious pathological conditions. However, the current way of diagnosing DVT is only possible after recognizing advanced DVT symptoms such as swelling, pain, and tightness in affected extremities, which may be due to the lack of information on neuromechanical changes following VC. Thus, the goal of this study was to investigate acute neuromechanical changes in muscle electrical activity and muscle stiffness when VC was induced. The eight pigs were selected and the change of muscle stiffness from the acceleration and muscle activity in terms of integral electromyography (IEMG) was investigated in three VC stages. Consequently, we discovered a significant increase in the change in muscle stiffness and IEMG from the baseline to the VC stages (*p* < 0.05). Our results and approach can enable early detection of pathological conditions associated with VC, which can be a basis for further developing early diagnostic tools for detecting VC-related diseases.

## Introduction

Venous congestion (VC) results from an imbalance between arterial inflow and venous outflow and can occur in the extremities due to reduced or obstructed venous flow [1]. This can lead to VC-related diseases such as deep vein thrombosis (DVT), varicose vein, venous ulcer, etc. [2–5]. DVT is associated with life-threatening disorders such as pulmonary thromboembolism, chronic thromboembolic pulmonary hypertension, and post-thrombotic syndrome [6]. Currently, the diagnosis of DVT involves venography or duplex ultrasound after clinical signs and symptoms of DVT appear, and can only detect the irreversible stage of DVT [2,7]. As a result, permanent damage to the extremities is inevitable, leading to a significant economic burden on patients and the healthcare system [8]. Additionally, recent studies have identified VTE as a fatal complication associated with COVID-19 [9].

Early identification of VC is crucial in protecting against permanent alternations of extremities, such as extremity pain, tightness, and swelling, that may result from VC-associated diseases like DVT. However, despite the importance of early detection of VC-associated diseases such as DVT, the underlying physiological changes related to this condition are not yet fully understood. Previous animal and human model studies have demonstrated endothelial and neurohormonal activation, as well as inflammation during experimental VC [10]. Extremity ischemia can lead to the deterioration of the peripheral nerve action potential [10], reduced muscle fiber conduction velocity, and decreased frequency shift of electromyography (EMG) during muscle contractions [11]. Furthermore, reduced blood flow to the muscles can hinder the removal of toxic metabolites, such as lactic acid, which may result in fatigue-induced EMG changes [11]. Similar to extremity ischemia, venous capillary or venule congestion in muscles, and consequent action potential change of muscle fibers, is one of the early physiological changes in VC [11]. These neuromechanical changes in the muscles can be measured using EMG for muscle electrical activity and acceleration (ACC) on the skin of the targeted muscle in the presence of mechanical impulses such as vibration for muscle stiffness [12]. However, there is a lack of information on neuromechanical changes due to VC being in a relaxed state. Understanding neuromechanical changes in a relaxed state due to VC can help us in developing an early diagnostic tool for VC-related diseases such as DVT.

Therefore, the goal of our study was to investigate whether VC in a relaxed state can result in acute neuromechanical changes in muscle electrical activity and muscle stiffness. We hypothesize that when the vibration was applied, there would be noticeable neuromechanical changes in muscle electrical activity and muscle stiffness due to venous congestion in a relaxed state. To validate our hypothesis, we performed experiments on anesthetized pigs’ legs. The pig leg model was chosen due to its close resemblance to the condition of completely obstructive symptomatic proximal DVT in a human limb [13]. Additionally, we induced VC in three stages to observe the progressive neuromechanical changes that occur during the progression of acute VC. In our study, the percentage of CIV blockage served as an indicator for inducing DVT.

## Materials and methods

### Subjects

Our study was approved by the Institutional Animal Care and Use Committee of the Korea University College of Medicine prior to the experiment (KOREA-2019-0101-C1). The study was conducted following the ARRIVE (Animal Research: Reporting of In Vivo Experiments) guidelines. After conducting a pilot study on the EMG and ACC at various time points, eight conditioned three-way (Landrace × Duroc × Yorkshire) crossbred pigs (8 males) were selected for our experiment. It is widely recognized that there are no significant differences between the venous systems of humans and pigs [14–16]. Each pig was housed comfortably in a cage with easy access to water and was allowed to rest for three days prior to the experiment, with the exception of the eight hours preceding it. At the conclusion of each experiment, each pig was euthanized, with an intravenous injection of potassium chloride (2 mmol/kg).

### Anesthesia and DVT surgery

Before anesthesia, sedation was done using the cervical intramuscular injection of Xylazine (2 mg/kg), Azaperone (5 mg/kg), Alfaxalone (4 mg/kg), and Atropine (0.05 mg/kg). Then, for fluid replacement, the intravenous catheter was placed on a dorsal ear and NaCl (0.9%) was infused. Xylazine (0.5 mg/kg) and Alfaxalone (1.5 mg/kg) were injected intravenously for inducing anesthesia. Then, deep anesthesia was maintained by inhaling isoflurane gas.

The central blood pressure was continuously monitored using a heparinized 24-gauge catheter (Jelco® IV catheter radiopaque, Smith Medical, UK) inserted in the carotid artery. The heparinized 24-gauge catheter was connected to a pressure-transducing device (AMK 150®, Ace Medical Co., Korea Rep.). On a monitor (BM5, Bionet Co. Berlin, Germany), the output of the pressure transducer was displayed. The pig’s vital signs were continuously monitored throughout the whole experiment.

**Fig 1:**
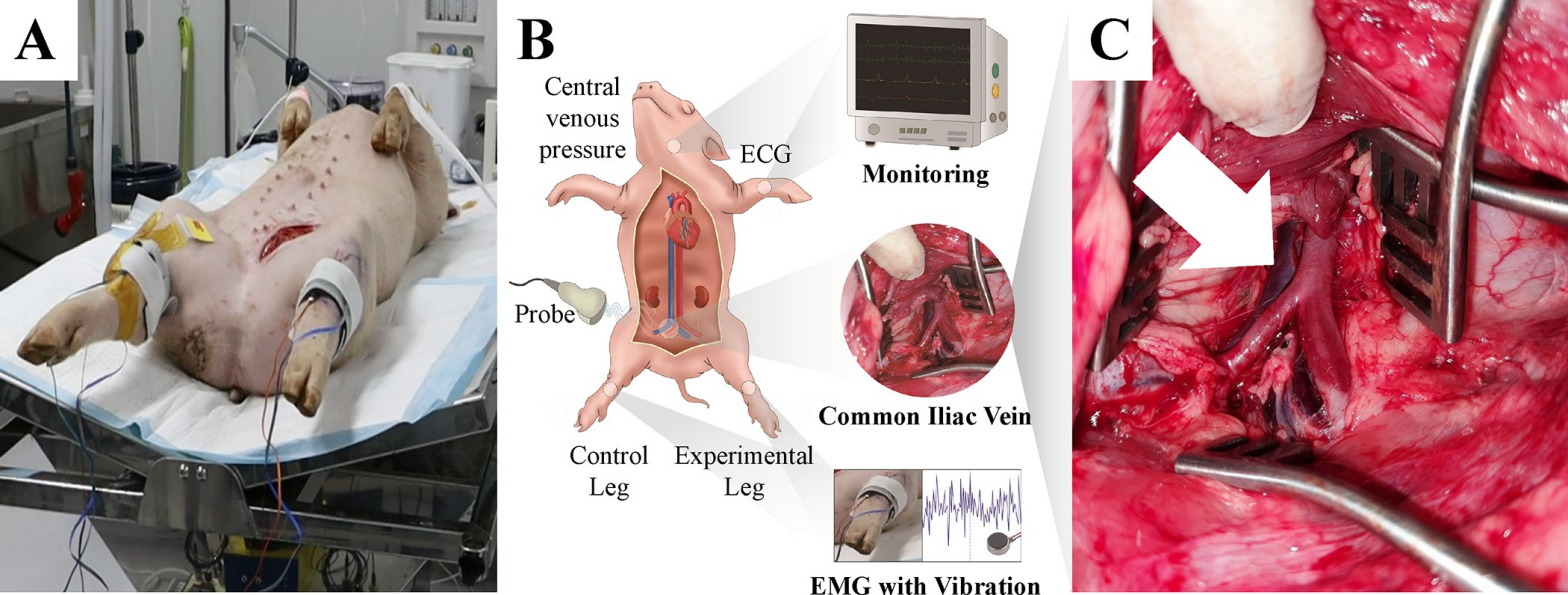
Experimental environment. (A) Each pig was placed in a supine position on a surgical table. (B) The vital signs were continuously monitored and the electromyography (EMG) electrode, accelerometer, and vibration motor were attached to the tibialis anterior muscle of the right (control) and left (experimental) hind limb using straps. (C) A common iliac vein was identified in the retropelvic space (white arrow) for the left hind limb of the pig.

Each pig was placed, in a supine position, on a surgical table (Fig 1A). A 15-cm-sized skin incision was made along with the left hind limb’s inguinal line. After deep dissection, the femoral vascular bundle was exposed between the adductor muscle and vastus medialis muscle. The femoral vein was carefully dissected after the incision of the inguinal ligament. In the retropelvic space, the common iliac vein (CIV) was identified (Fig 1C).

### Data acquisition

For data acquisition, an EMG electrode (13E202, Ottobock, Germany), an accelerometer (352A71, PCB Piezotronics Inc., USA), and a cylinder-type vibration motor (DVM7, D&J WITH Co., Ltd., Korea) were attached to the tibialis anterior muscle of the right (control) and left (experimental) hind limb using the straps, respectively (Fig 1A). The rated voltage and rated current of the vibration motor were 3V and 60 mA, respectively. The max-rated rotation was 11,000 ± 1,500 rpm and the weight was 30 g. The EMG and ACC data were acquired at 1,000 Hz using a customized Labview program (National Instruments, Austin, TX, United States).

**Fig 2:**
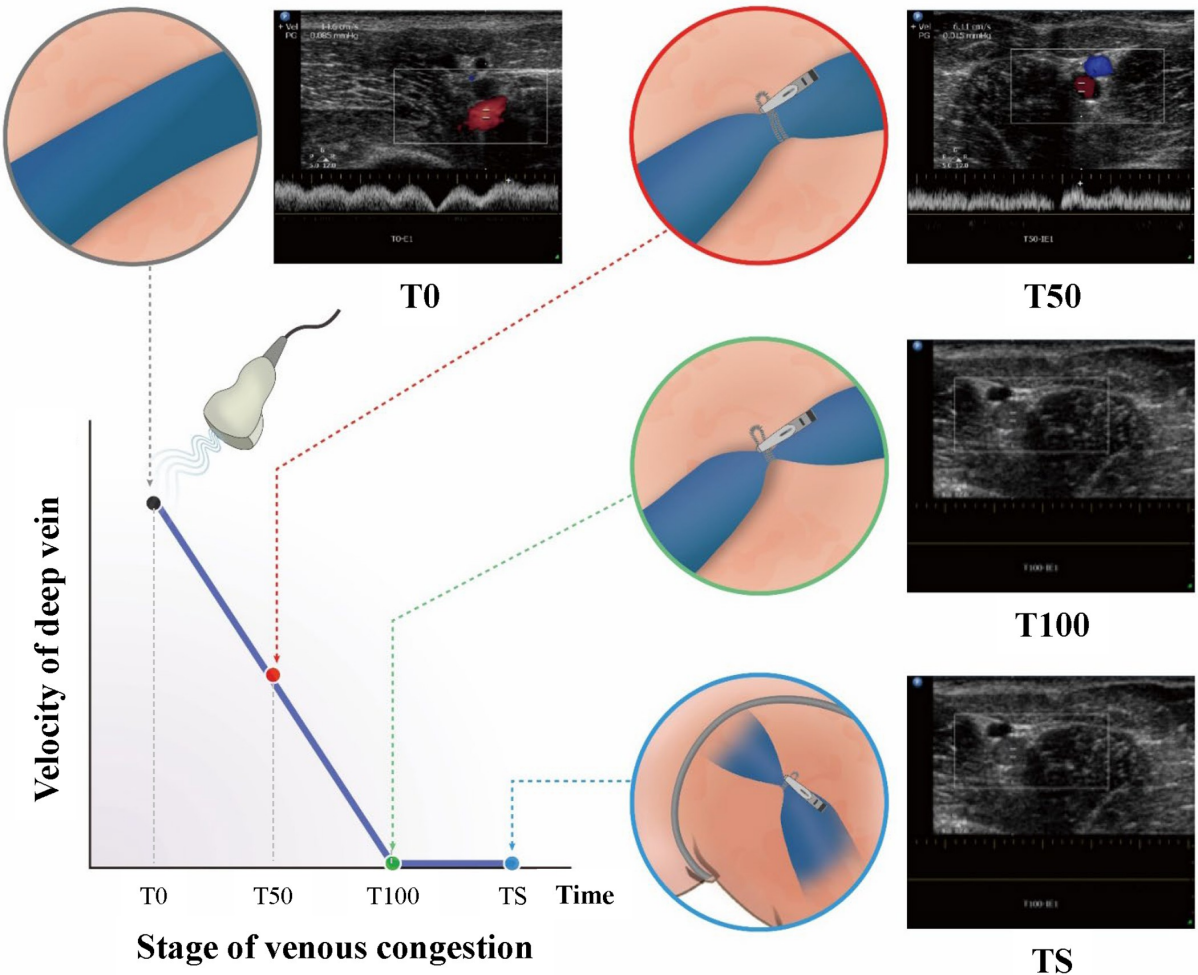
The common iliac vein was tied using a rubber band to control the venous flow velocity (50% and 100% of baseline venous velocity). Lastly, the proximal thigh was also tied with an elastic band to block superficial veins around the inguinal area.

**Fig 3:**
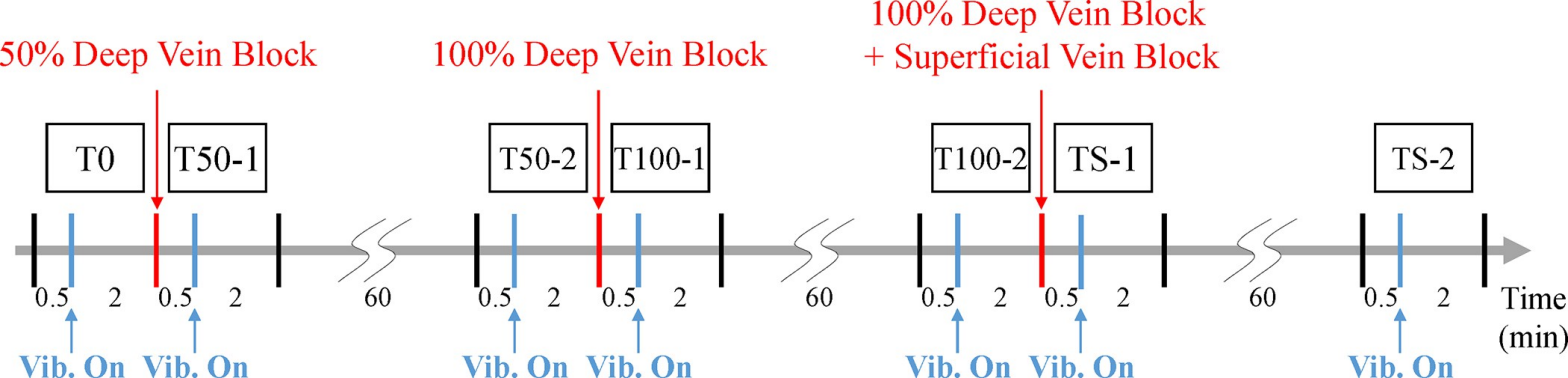
Experimental protocol. The EMG and acceleration data were acquired at 1,000 Hz, immediately and 1 hour after venous flow control.

To observe the gradual neuromechanical changes during acute VC progression, we implemented a phased approach in inducing VC using a rubber sling to block venous flow (Fig 2) in the common iliac vein (CIV) of each pig. This phased ligation of the CIV aimed to simulate VC similar to that observed in DVT. Prior to the surgical procedure, baseline measurements (T0) of the EMG and ACC were obtained using EMG electrodes, accelerometers, and an ultrasonic device. Data were collected both without vibration (30 sec) and with vibration (2 min). Subsequently, VC was induced by partially blocking the venous flow of the CIV by 50% (T50). To verify the presence of VC in the experimental legs, Doppler ultrasonography was employed to assess venous flow and confirm the extent of blockage at 50% and 100% levels. These measurements were then compared to those obtained from the control legs, serving as a reference (Fig 2). EMG and ACC data without vibration (30 sec) and with vibration (2 min) were acquired, immediately after the surgery at the 50% blockage stage (T50-1) and 1 hour later (T50-2), respectively. Following that, the venous flow of the CIV was completely blocked (T100), and corresponding EMG and ACC data without vibration (30 sec) and with vibration (2 min) were collected immediately after achieving 100% blockage (T100-1) and 1 hour later (T100-2), respectively. Finally, to simulate the induction of complete obstruction seen in DVT, the superficial veins around the thigh were occluded. Similarly, EMG and ACC data without vibration (30 sec) and with vibration (2 min) were obtained, immediately after tying the proximal thigh with an elastic band (TS-1) and 1 hour after (TS-2) (Fig 3).

### Data analysis

EMG and ACC data were analyzed to investigate the mechanical and neuromuscular effects induced by the vibration across all VC stages. The mechanical and neuromuscular effects were investigated using integrated EMG (IEMG) and muscle stiffness used in previous studies [17–20]. The collected EMG data were rectified and filtered by proprietary onboard that produces outputs appropriate for graded control [21] with the high common mode rejection in the low-frequency range (>100 dB at 50 Hz). The EMG data were normalized to peak EMG occurring across all of the VC stages at each experimental leg and control leg. For extracting EMG features, a window size of 256 msec with an overlap of 128 msec was used [22]. IEMG can show the changes in EMG signal strength over time. It was computed as the sum of the area under the curve of the signal over a given time window as seen in Eq (1):

$$IEMG=\sum_{i=1}^{N}\left|x_{i}\right|$$ (1)

where *i* and *N* denote the window length and *x*_*i*_ is the EMG data within the given window [23]. Then delta IEMG, ΔIEMG at each VC stage, was computed by subtracting the median IEMG at the baseline.

Mechanical properties of muscle can be investigated using the stiffness measure, which can be an objective indicator of physiological characteristics such as muscle pain, fatigue, and cramps [24,25]. Stiffness was computed from the ACC data based on previous studies using a Myotonometric device [26]. The ACC data from the sensor was band-pass filtered between 75 and 160 Hz to remove noise and artifacts. The filtered ACC data were then detrended and double-integrated to get the muscle deformation depth position/displacement. A time window of 1 sec was used to compute the muscle stiffness at each VC stage. Muscle stiffness is the muscle tissue’s resistance to an external force caused by the vibrating unit [25–27]. It was calculated using the following Eq (2):

$$Muscle\;Stiffness=m*a_{max}/\Delta l$$ (2)

*a*_*max*_ is the maximum acceleration within the 1-second window, whereas *m* refers to the mass of the vibrating unit, and *Δl* refers to the deformation depth of the muscle at maximum acceleration obtained from double integration of the ACC data [28]. Next, Δmuscle stiffness was calculated by subtracting the median stiffness value at the baseline from each VC stage of both the experiment and control legs.

The median of ΔIEMG at each VC stage was used for statistical group comparison in both vibration and without vibration conditions. The normality of baseline (T0) and each of the VC stages (T50-1~TS-2) was checked by the Kolmogorov-Smirnov test. Then, the Mann-Whitney U test at each time point was applied to investigate differences between the baseline and each VC stage. Similarly, the differences between the experiment and control legs were checked independently at each VC stage. The same approach was used to investigate the differences in delta muscle stiffness across the legs at each VC stage and between baseline and each VC stage for both legs.

## Experimental results

**Fig 4:**
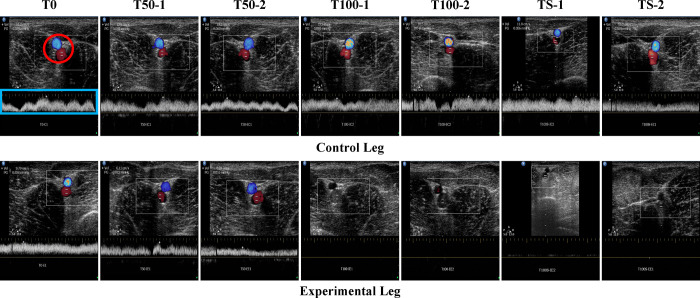
The representative ultrasound images in each VC stage in the experimental leg. The red circle represents the common iliac vascular bundle, and the white graph (in the blue rectangle) the venous flow in the red circle.

To confirm the presence of VC during our experiment, we used duplex ultrasound imaging to examine the venous flow of the CIV (Fig 4). The baseline image (T0) showed the common iliac vascular bundle and venous flow in the control leg (Fig 4). VC was then induced by blocking the venous flow of the CIV in the experimental leg by 50% and 100%, respectively, and was compared with the baseline venous velocity (Fig 4).

**Fig 5:**
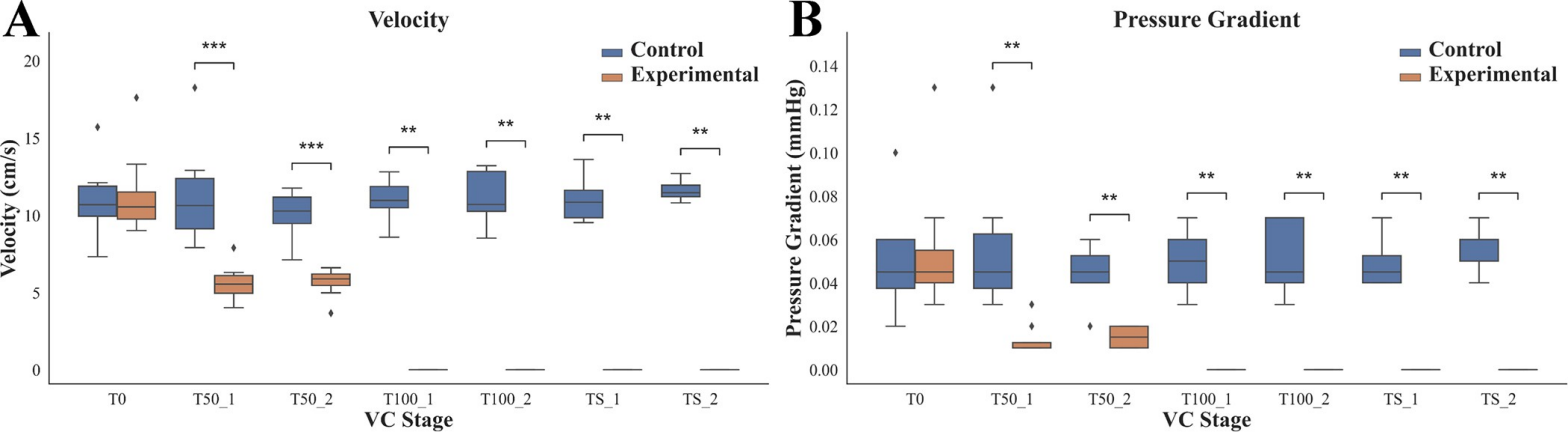
Results in maximum velocity of CIV and pressure gradient at each stage related to VC of the pig legs. (A) The maximum velocities of the venous flow of the CIV between the control and experimental legs. (B) The pressure gradient between the control and experimental legs. Each box plot indicates the 25^th^ (Q1), 50^th^ (Q2), and 75^th^ (Q3) percentiles of the data (**p < 0.01, and ***p < 0.001). The whisker indicates Q3 + 1.5 × (Q3 –Q1), and Q1 − 1.5 × (Q3 –Q1) and the dot indicates the outliers.

Fig 5 displays the maximum velocity (Fig 5A) and pressure gradient (Fig 5B) of the venous flow of the CIV in the control and experimental legs of all pigs. At T0, there was no significant difference in velocities between the control and experimental legs (Fig 5A). After blocking venous flow (T50-1, T50-2, T100-1, T100-2, TS-1, and TS-2), VC was sufficiently achieved in each stage (see Fig 5A). There were significant differences (*p* < 0.01) in the velocities at each stage between the control and experimental legs. Similarly, there was no significant statistical difference in the pressure gradient at baseline (T0) between the control and experimental legs, but after VC induction, there were significant differences (*p* < 0.01) in the pressure gradients at each stage.

**Fig 6:**
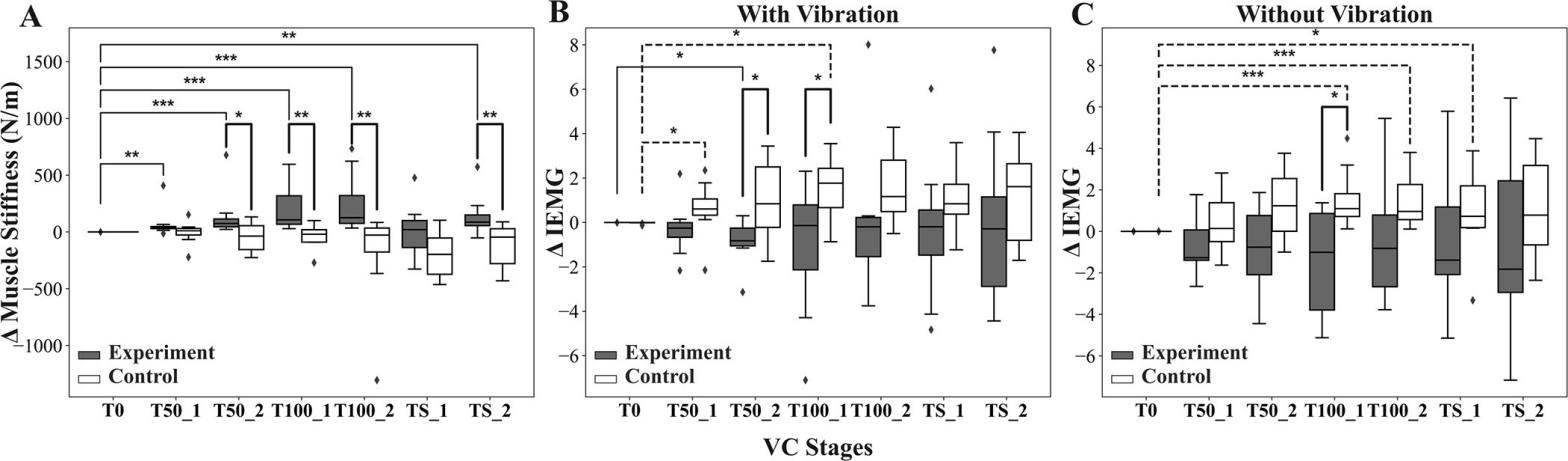
Changes in muscle stiffness and muscle electrical activity between the experimental and control legs. (A) The changes in muscle stiffness across all VC stages in the experimental and control leg. The bold dark lines indicate the statistical differences between the experimental and control legs, whereas the solid lines indicate differences between the baseline (T0) and each VC stage in the experimental leg. The broken lines indicate the differences between the baseline and each of the VC stages in the control leg. (B) The changes in ΔIEMG (integral electromyography) across all VC stages with vibration. (C) The changes in ΔIEMG across all VC stages without vibration (*p < 0.05, **p < 0.01, and ***p < 0.001).

Fig 6 shows the changes in muscle stiffness, estimated using ACC signals and muscle electrical activity from EMG, between the experimental and control legs. The experimental leg showed a significantly greater increase in muscle stiffness change from baseline (Δmuscle stiffness) compared to the control leg (see Fig 6A). There were statistically significant differences in the change in Δmuscle stiffness between the experiment and control legs (*p* < 0.05) at T50-2, T100-1, T100-2, and TS-2. Additionally, a statistically significant difference in Δmuscle stiffness between the baseline and each of the VC stages was observed in the experimental leg. In contrast, no statistically significant differences in Δmuscle stiffness were observed between baseline and any of the VC stages in the control leg.

Furthermore, the changes in integral EMG (IEMG) at each VC stage from baseline (ΔIEMG) with vibration were greater in the control leg than in the experimental leg (see Fig 6B). The group comparison of ΔIEMG between the experimental and control legs showed a statistically significant difference (*p* < 0.05) at the T50-2 and T100-1 VC stages. Within the experimental leg, the VC stage T50-2 showed a significant difference (*p* < 0.05) in ΔIEMG compared to the baseline, while within the control leg, the VC stages T50-1 and T100-1 showed significant differences (*p* < 0.05) in ΔIEMG compared to the baseline.

Similarly, ΔIEMG without vibration was higher in the control leg than in the experimental leg (see Fig 6C). However, only VC stage T100-1 showed a statistically significant difference (*p* < 0.05) in ΔIEMG between the experimental and control legs. Within the experimental leg, there was no significant difference between the VC stages and baseline. However, within the control leg, there was a significant difference between the baseline and VC stages T100-1 (*p* < 0.001), T100-2 (*p* < 0.001), and TS-1 (*p* < 0.05).

## Discussions

In this study, significant neuromuscular and mechanical changes were observed in muscles among different VC stages in the pig model. The VC induced by the phased ligation of the CIV closely mimics the VC observed in cases of DVT. In order to achieve different VC stages, a rubber sling was tied to block the venous flow in the experiment. To measure changes in EMG and ACC under vibrotactile stimulation, EMG electrode, accelerometer, and vibration motor were attached to the tibialis anterior muscle of both hind limbs using straps. However, the straps used in this research setup prevented the measurement of intramuscular pressure and muscle stiffness as shear elastic modulus, simultaneously. In our previous study, it was shown that muscle compartment syndrome increased intramuscular pressure and influenced muscle stiffness using shear-wave elastography [29]. Therefore, the change in the difference in derived muscle stiffness from the ACC data (Fig 6A) between the control and experimental legs indicates that mechanical changes associated with VC could occur.

A previous study [30] has indicated that muscle swelling can be caused by various factors, including 1) venous capillary congestion, 2) increased interstitial fluid, 3) enlargement of individual fibers, 4) interfibrillar edema, and 5) edema of the perimysium. Approximately 1 hour of experimental venous congestion has been shown to be adequate for inducing these physiologic changes in both large animals and humans [10,31–33]. Thus, we devised an experimental protocol that involved phased blockade of venous flow at 1-hour intervals. Our results indicate that the increased muscle stiffness observed in Fig 6A may be attributed to muscle swelling caused by VC. Additionally, in the cases of the ΔIEMG (Fig 6B and 6C), some changes were observed between the experimental and control legs, but statistically significant differences were only observed in a few VC stages. It appeared that the variation of ΔIEMG was larger in the experimental leg, particularly without vibrotactile stimulation, compared to the condition with vibrotactile stimulation. The physiological events associated with VC stages in the experimental leg can change EMG signals. It can be interpreted that when the venous flow is partially or completely blocked, the soft tissue would swell and become stiff due to VC. Subsequently, VC would result in increased intracompartmental pressure and muscle stiffness. Finally, the increased compartment pressure would cause decreased tissue perfusion pressure and muscle ischemia, leading to changes in the EMG signal [34–37]. However, our experiment was only conducted under anesthetic conditions, additional experiments are needed to investigate changes in muscle activation due to VC under non-anesthetic conditions.

Based on the experimental results, we found for the first time that there are significant neuromechanical changes in muscle electrical activity and muscle stiffness resulting from venous congestion in a relaxed state. Our finding on our experimental results is subtle changes in muscle stiffness and ΔIEMG (Fig 6A, 6B and 6C) originating from skeletal muscle [38] associated with vibration stimuli. Although other pathologic conditions that cause muscle swelling like myositis, intramuscular hemorrhage, and muscle injury might not be separated; however, their distinctive clinical signs, symptoms, and medical history or trauma will not cause misdiagnosis of VC [39]. Therefore, our findings have valuable clinical implications for monitoring the development of venous congestion-related diseases, including DVT, varicose veins, venous ulcers, and more. Moreover, these findings serve as an important warning sign for clinicians, alerting them to the current occurrence of venous congestion-related diseases.

Regarding the vibration used in our study, it has similar power to that of a commercial cellular phone. The vibrating stimulus was just used to characterize muscle stiffness, not for therapeutic purposes. In our previous study [13], we investigated to determine how deep the vibration can be transmitted from a vibration motor attached to the thigh skin of pigs. The acceleration data was acquired at four randomized points in the thigh muscles to examine the impact of vibration stimulation on vibration transmission depending on the depth of the soft tissue. The depth was measured using an ultrasound machine. On average, the vibrations were detected at a depth of 3.06±0.08 cm beneath the skin, and were not detectable at a depth of 3.89±0.08 cm beneath the skin [40]. When considering the application of vibration to the human calf, which is the most susceptible area for human DVT, a transmission depth of 3~4 cm is sufficient to reach the calf muscles and is shallower than the deep veins [41]. Thereby, our approach could not directly cause thrombus shedding. In further research, the effect of vibration frequency and amplitude on thrombus shedding will be studied in humans.

## Conclusion

Our study’s results, which measured muscle stiffness and ΔIEMG, supported our hypothesis of significant changes in the neuromuscular and mechanical characteristics of pig muscles inducted by VC. The significant increase in the change in muscle stiffness from the baseline to the VC stages in the experimental leg, compared to the control leg, could indicate a physiological response to the induced VC. Therefore, our approach can be useful in monitoring and detecting various pathological conditions such as VTE, DVT, venous ulcers, varicose veins, and other venous insufficiencies before the appearance of symptoms such as swelling and tightness. Early detection can improve patient survival and prevent permanent alterations of the extremities.

## Supporting information

S1 File The raw data used to build the graphs (Fig 6). (XLSX)
