# Correction: Venous congestion affects neuromuscular changes in pigs in terms of muscle electrical activity and muscle stiffness

**DOI:** 10.1371/journal.pone.0316652

**Published:** 2024-12-26

**Authors:** Keun-Tae Kim, Duguma T. Gemechu, Eunyoung Seo, Taehoon Lee, Jong Woong Park, Inchan Youn, Jong Woo Kang, Song Joo Lee

There are errors in the author affiliations. The correct affiliations are as follows:

Keun-Tae Kim^1^, Duguma T. Gemechu^1,2^, Eunyoung Seo^1^, Taehoon Lee^1^, Jong Woong Park^3^, Inchan Youn^1^, Jong Woo Kang^4^, Song Joo Lee^1,2^

**1** Korea Institute of Science and Technology, Bionics Research Center, Seoul, Korea Repub, **2** Division of Bio-Medical Science & Technology, KIST School, Korea University of Science and Technology, Seoul, Korea Repub, **3** Department of Orthopaedic Surgery, Korea University Anam Hospital, Seoul, Korea Repub, **4** Department of Orthopaedic Surgery, Korea University Ansan Hospital, Ansan, Korea Repub.
